# SGLT2 Inhibitors: A Broad Impact Therapeutic Option for the Nephrologist

**DOI:** 10.3389/fneph.2022.867075

**Published:** 2022-04-29

**Authors:** Antonio Granata, Francesco Pesce, Massimo Iacoviello, Massimiliano Anzaldi, Francesco Amico, Maria Catalano, Giuseppe Leonardi, Carmela Gatta, Giusy Costanza, Salvatore Corrao, Loreto Gesualdo

**Affiliations:** ^1^ Nephrology and Dialysis Unit, “Cannizzaro” Emergency Hospital, Catania, Italy; ^2^ Renal, Dialysis and Transplantation Unit, Department of Emergency and Organ Transplantation, University of Bari, Bari, Italy; ^3^ Cardiology Unit, Department of Medical and Surgical Science, University of Foggia, Foggia, Italy; ^4^ Diabetes Unit, “Cannizzaro” Emergency Hospital, Catania, Italy; ^5^ Cardiology Unit, “Cannizzaro” Emergency Hospital, Catania, Italy; ^6^ Cardiology Unit, Azienda Ospedaliera Universitaria (A.O.U.) “Policlinico-San Marco”, Catania, Italy; ^7^ Internal Medicine Unit, Azienda Ospedaliera Universitaria (A.O.U.) “Policlinico-San Marco”, Catania, Italy; ^8^ Nephrology and Dialysis, “Vittorio Emanuele” Hospital, Gela, Italy; ^9^ Department of Internal Medicine, “Azienda di Rilievo Nazionale ed Alta Specializzazione (ARNAS) Civico, Di Cristina e Benfratelli”, Palermo, Italy

**Keywords:** SGLT 2 inhibitors, diabetes, CKD - chronic kidney disease, glycemic control, Cardioprotection, Nephro- protection

## Abstract

Since their introduction as antidiabetic drugs, SGLT2 inhibitors (SGLT2i) have come a long way, proving to be beneficial on cardiovascular and renal outcomes independently of diabetes status. The benefits go far beyond glycemic control, and both the cardio- and nephroprotection are underpinned by diverse mechanisms. From the activation of tubule glomerular feedback and the consequent reduction in hyperfiltration to the improvement of hypoxia and oxidative stress in the renal cortex, SGLT2i have also been shown to inhibit hepcidin and limit podocyte damage. Likewise, they improve cardiac metabolism and bioenergetics, and reduce necrosis and cardiac fibrosis and the production of adipokines, cytokines, and epicardial adipose tissue mass. In terms of outcomes, the efficacy has been demonstrated on blood pressure control, BMI, albuminuria, stroke, heart disease, and mortality rate due to cardiovascular events. Patients with chronic kidney disease and proteinuria, with or without diabetes, treated with some SGLT2i have a reduced risk of progression. The analysis of subgroups of individuals with specific diseases such as IgA nephropathy has confirmed this solid effect on renal outcomes. Given these overarching activities on such a broad pathophysiological background and the favorable safety profile that goes with the use of SGLT2i, it is now certain that they are changing our approach to clinical interventions for important outcomes with an impressive impact.

## Introduction

Diabetic kidney disease (DKD) is a microvascular complication associated with at least 30% of the diabetes cases worldwide and a well-established leading cause of chronic kidney diseases (CKDs) and the end-stage renal disease (ESRD) (1).

DKD is a global burden, and in 2012, its mortality rose by 94% (2). DKD risk factors can be classified into three major categories: susceptibility risk factors (such as age, sex, family history, ethnicity), initiation factors (such as hyperglycemia), and progression factors (such as hypertension, diet, obesity) (3).

DKD is associated with numerous structural alterations in the kidney, as reported by different authors. Most common structural alterations include an increased thickness in different kidney compartments, such as glomerular, capillary, tubular, and basement membrane (4–6). Moreover, the pathophysiology of DKD consists of different critical metabolic changes, which promote inflammation and fibrosis. These changes include hyper aminoacidemia, glomerular hyperfiltration, glomerular hyper infusion, and hyperglycemia (7–9).

Diagnosis of DKD is mostly clinical but also includes the measurement of eGFR and albuminuria. In fact, during DKD, blood tests for kidney function result in a persistent high urinary albumin/creatinine ratio (≥30 mg/g) and a reduction in eGFR (<60 ml/min per 1.73 m^2^) (10). However, Klessens et al. conducted a study, which revealed that both proteinuria and a reduced eGFR failed in the DKD diagnosis (11).

Unfortunately, long-term therapies aimed at glycemic control are not sufficient to reduce the DKD risk. Nowadays, new therapeutic strategies aim to reduce glomerular hyperfiltration, inflammation, and fibrosis (12–15). Sodium-glucose cotransporters type 2 inhibitors (SGLT2i) have been suggested as a novel, insulin-independent therapeutic approach for managing T2DM (16).

## Renal Sodium-Glucose Cotransporters and Their Inhibition

The kidney plays an important role in the maintenance of glucose homeostasis. In fact, it is involved in gluconeogenesis and glucose reabsorption at the glomerular level. Glucose is a polar molecule, able to cross the cell membrane from the lumen to the tubule through an active transport mediated by two sodium/glucose carriers, called SGLT2 and SGLT1. SGLT2 is located in the first segment of the proximal tubule, and it is responsible for the reabsorption of 90% of the filtered glucose. On the contrary, SGLT1 is located in the third segment of the proximal tubule, and it is responsible for the reabsorption of the remaining 10%. The filtered glucose is totally reabsorbed into the tubule, reaching a maximal reabsorption threshold corresponding to 180–200 mg/dl of blood glucose (17–19).

When the blood glucose levels are above the threshold, the system capacity will be exceeded, and glucose in the urine will be observed (20).

In diabetic patients, in a condition of hyperglycemia, the amount of glucose filtering into the glomerulus is enhanced, leading to a condition called glomerular hyperfiltration (21).

Glomerular hyperfiltration consists of an alteration of the early stages of the CKD in both T2DM and T1DM (22). However, glomerular hyperfiltration is not always the leading cause of DKD (23). SGLT2i belong to the gliflozins class and had initially been developed to improve glycemic compensation in T2DM patients. In the last years, the rationale for using this class of drugs has changed due to the emerging favorable effects on cardiovascular (CV) and kidney outcomes (24).

SGLT2i mechanism of action is insulin independent (25–28) and acts by blocking the filtered glucose reabsorption and increasing urinary glucose excretion (29–31).

SGLT2i can be administered as monotherapy or in combination with other classes of glucose-lowering drugs (16).

So far, numerous SGLT2i have been studied. To date, the available formulations are classified according to their selectivity for the SGLT2/1 transporters. Selective compounds include dapagliflozin, empagliflozin, and ertugliflozin, which are involved in the exclusive inhibition of the SGLT2 transporter. On the other hand, canagliflozin and sotagliflozin present an inhibitory effect even on SGLT1 (32). To date, four different oral formulations have been approved in the US and many more regions, including European Union (EU) and Japan for daily administration [alone or in combination with metformin or dipeptidyl peptidase 4 (DPP4)] (**Table 1**) (24).

**Table 1:** SGLT2i approved by the European Medicines Agency.

Drug	Association	Commercial name	Dosage
**Empaglifozin**	/	Jardiance^©^	10 mg
			25 mg
	Metformin	Synjardy^©^	5 mg + 850 mg
			12.5 + 850 mg
			5 + 1,000 mg
			12.5 + 850 mg
	Linagliptin	Glyxambi^©^	10 mg + 5 mg
			25 mg + 5 mg
**Canaglifozin**	/	Invokana^©^	100 mg
	Metformin	Vokanamet^©^	50 + 850 mg
			150 + 1,000 mg
			50 + 1,000 mg
			150 + 850 mg
**Dapaglifozin**	/	Forxiga^©^	10 mg
	Metformin	Xigduo^©^	5 + 850 mg
			5 + 1000 mg
	Saxagliptin	Qtem^©^	5 mg + 10 mg
Ertugliflozin	/	Steglatro^©^	5 mg
			15 mg
	Metformin	Segluromet^©^	2.5 mg + 850 mg
			2.5 mg + 1,000 mg
			7.5 mg + 850 mg
			7.5 mg + 1,000 mg
	Sitagliptin	Steglujan^©^	5 mg + 100 mg
			15 mg + 100 mg

[Adapted from Costanza et al., 2020 (24)].

Various registrative trials have been conducted to evaluate the safety of these molecules. Interestingly, they demonstrated to be not only safe but also able to significantly reduce major CV events, hospitalizations, and the exitus due to CV events (24).

Three clinical trials have been conducted to evaluate the CV safety of SGLT2i: EMPA-REG OUTCOME, CANVAS, and DECLARE TIMI 58. In total, 34,322 patients were included. The EMPA-REG OUTCOME enrolled patients with 0% of multiple risk factors and 100% previous CV event, while this proportion rose in the CANVAS program (34%) and in the DECLARE TIMI 58 (59%). Diabetic patients with a glomerular filtration rate (GFR) <60 ml/min/1.73 m^2^ (25.9% in the EMPA-REG OUTCOME, 20.1% in the CANVAS program and 7.4% in the DECLARE TIMI 58) have been recruited. In particular, the EMPA-REG OUTCOME included CV benefits in patients with a GFR of 30–60 ml/min/1.73 m^2^. Results showed that patients treated with SGLT2i presented a reduced risk of CV events than the placebo group. In particular, SGLT2i reduced the establishment of major CV events by 11%, as well as the CV death and hospitalizations (33). The relative risk reduction ranged between 27% and 35% (34). Moreover, some benefits for the kidney have been registered (33).

These results encouraged new retrospective studies and new clinical trials to evaluate the protective effect of SGLT2i on the kidney. In particular, Toyama et al. conducted a metanalysis including 27 studies and 7,363 diabetic patients. SGLT2i demonstrated to be involved in the improvement of numerous outcomes, such as blood pressure control, BMI and albuminuria reduction, CV risk reduction, stroke, and heart disease reduction, as well as a decrease in the mortality rate due to CV events. Moreover, SGLT2i were safe and well tolerated (35).

The DAPA CKD study confirmed the efficacy of dapagliflozin to reduce the risk of sustained decline in the eGFR with a hazard ratio of 0.56 (95% CI, 0.45 to 0.68; *p* < 0.001), end-stage kidney disease, and death from renal or cardiovascular causes in a broad population, regardless of the presence or absence of diabetes (36).

## SGLT2i Nephroprotection

Current evidence suggests that numerous mechanisms can contribute to establishing kidney protection by SGLT2i. These mechanisms can be direct or indirect, as well as local or systemic. The first mechanism consists of glycemic control. In fact, SGLT2i promote glucose elimination with urine, but they also improve the use of glucose in the liver and reduce insulin resistance at the muscle level (37, 38). To evaluate this aspect, numerous studies have been conducted. In 2013, Vasilakou et al. conducted a systematic review and meta-analysis to assess the efficacy and safety of SGLT2i in adults with T2DM. Results demonstrated a reduction of HBA1c of 0.66% in patients with T2DM (39).

Moreover, Heerspink et al. reported that GFR decline was slowed down and that the proteinuria was reduced in T2DM patients treated with SGLT2i (40).

Overall, these data suggest that improved glycemic compensation certainly contributes but cannot alone explain the effectiveness of SGLT2i in kidney protection. These elements support the multifactorial nature of nephroprotection from SGLT2i, but, on the other hand, it opens new horizons on the possible effectiveness of these molecules in non-diabetic kidney disease. **Table 2** shows the main clinical studies conducted with SGLT2i, which have revealed pleiotropic effects of great interest in terms of kidney protection. Due to the limited correlation between clinical benefits from SGLT2i and glycemic control, the most recent work shows growing attention to the favorable effects of gliflozins even in the absence of DM.

**Table 2:** Main clinical studies involving SGLT2i.

Trial	Type of Study	Population	Drug	Main results
**EMPAREG OUTCOME**	Trial	7,020 patients with T2DM	Empaglifozin	↓ CV-related death↓hospitalization↓ death for any cause
**CANVAS**	Trial	10,142 patients with T2DM and high CV risk	Canaglifozin	↓ composite endpoint CV death, non-fatal myocardial infarction or stroke
**CREDENCE**	Trial	4,401 patients with T2DM, CKD, and microalbuminuria	Canaglifozin	↓ kidney composite endpoint↓ ESKD, GFR decline, albuminuria↓ hospitalization↓ composite endpoint CV death, non-fatal myocardial infarction or stroke.
**Yagi et al.** (41)	Observational	13 patients with con T2DM	Canaglifozin	↓ pericardial fat
**Sato et al.** (42)	Observational	40 patients with T2DM and coronopathy	Dapagliflozin	↓ pericardial fat, body weight, tumor necrosis factor alpha (TNF-α), plasminogen activator inhibitor type 1 (PAI-1)
**Bouchi et al.** (43)	Pilot	19 patients with T2DM	Luseogliflozin	↓ pericardial fat, body weight, BMI, blood pressure, triglycerides, RCP
**Heerspink et al.** (40) **Blonde et al.** (44)	Trial	1,450 patients with T2DM	Canaglifozin	↓ GFR decline↓albuminuria in patients with microalbuminuria and macroalbuminuria↓ BMI, body weight
**McGurnaghan et al.** (45)	Observational	8,566 pz con DM2	Dapagliflozin	↓ body weight BMI, blood pressure
**Kario et al.** (46)	Trial	132 patients with T2DM and hypertension	Empaglifozin	↓ blood pressure, body weight
**Solini et al.** (47)	Trial	40 patients with T2DM	Dapagliflozin	↓ blood vessels stiffness
**DAPA-HF**	Trial	4,744 patients with heart failure with and without T2DM	Dapagliflozin	↓ worsening heart failure or CV death with and without T2DM
**DAPA-CKD**	Trial	4,245 patients with CKD and microalbuminuria, with and without T2DM	Dapagliflozin	↓ composite endpoint of eGFR decline ≥50%, ESKD and CV or renal death
**DECLARE TIMI-58**	Trial	17,160 patients with T2DM and patients con DM2, high-risk or with atherosclerotic vasculopathy	Dapagliflozin	↓ composite endpoint CV death↓ hospitalization for heart failure
VERTIS	Trial	8,246 patients with T2D and established atherosclerotic cardiovascular disease	Ertugliflozin	Noninferior for CV, trends beneficial effect on renal outcomes

[Adapted from Costanza, 2020 (24)] ↓: reduction.

Furthermore, SGLT2i are characterized by various systemic mechanisms involved in kidney protection and are unrelated to glycemic control. In fact, SGLT2i can contribute to the weight and BMI reduction (44, 48) since the first dose (49).

SGLT2i can modulate the activation of the tubuloglomerular feedback through the increase in sodium delivery to the macula densa. This reduces the afferent arteriole vasodilatation, glomerular hypertension and the subsequent albuminuria (50). SGLT2i could also exert, by glycosuria, a diuretic osmotic effect (51–53) without inducing renin–angiotensin system activation. On the contrary, the increased delivery of sodium at the level of macula densa is associated with the activation of tubule-glomerular feedback and the consequent vasoconstriction of the glomerular afferent arteriole and the reduction of renin activity (54). In addition, the increased elimination of sodium and water acts in the blood pressure control and the cardiac load reduction (55). The mechanisms involved in the blood pressure reduction include the diuretic action (56), as well as the bodyweight and total sodium reduction (57). The reduction in the stiffness of the blood vessels might also play a role (58).

Several hypotheses have been postulated to explain the cardioprotective role of SGLT2i. Firstly, SGLT2i might induce a reduction in preloading and after loading conditions through the osmotic diuresis, reducing blood pressure and improving CV function. Other mechanisms might involve the improvement of cardiac metabolism and bioenergetics, the inhibition of the myocardial Na+/H+ exchange, the reduction of necrosis and cardiac fibrosis, and the alteration in the production of adipokines, cytokines, and epicardial adipose tissue mass (51).

Moreover, according to some animal model studies, SGLT2i might also play an anti-inflammatory role. In fact, SGLT2i demonstrated to be involved in reducing inflammatory markers production in the kidney (59). Finally, other favorable effects have been hypothesized at the cardiac level (51). SGLT2i could improve the myocardial energetics by increasing hematocrit (60) and oxygen delivery as well as the changes of energetic substrates (61). In fact, SGLT2i are able to fix the imbalance between oxygen demand and oxygen supply that is commonly seen in diabetic patients.

The increase in hematocrit may be due to EPO upregulation induced by intensified hypoxia at the renal cortico-medullary junction (62). In addition, the inhibition of hepcidin by SGLT2i may increase iron bioavailability and utilization (63).

There is evidence that SGLT2i limits podocyte damage (64), and the efficacy in glomerulonephritis is becoming apparent, as dapagliflozin has been shown to reduce the risk of CKD progression in patients with IgA nephropathy (65).

The most important beneficial effects of SGLT2i are reported in **Table 3**.

**Table 3:** Most important beneficial effects of SGLT2i.

Most important beneficial effects of SGLT2i
Increased glycemic control
Reduction of afferent arteriole vasodilatation
Reduction of glomerular hypertension
Reduction of albuminuria
Increase of Na elimination
Reduction of blood pressure
Increased hematocrit and oxygen delivery
Reduction of preloading and after-loading condition
Reduction of myocardial Na/H exchange
Increased cardiac metabolism
Reduction of epicardial adipose tissue mass
Reduction of necrosis and cardiac fibrosis
Reduction of epicardial adipose tissue mass
Decrease in production of adipokines and cytokines
Weight loss
BMI reduction
Anti-inflammatory activity

In summary, SGLT2i seem to act on multiple systemic aspects, known CV risk factors, and CKD development and progression. Similarly, the local mechanisms underlying nephroprotection also appear to be manifold. De Nicola et al. have suggested that SGLT2i can counteract glomerular hyperfiltration, regardless of the hypoglycemic effect, while having benefits related to albuminuria, inflammation, and nephromegaly secondary to the improvement of glycemic control (66). The effects of SGLT2i will certainly be investigated in depth in large cohorts of patients now that FDA and EMA have granted the approval of dapagliflozin to reduce the risk of kidney function decline, kidney failure, cardiovascular death, and hospitalization for heart failure in adults with CKD (67).

## SGLT2i Kidney Protection and Cardiovascular Outcomes in Type 2 Diabetes

As confirmed by a lot of evidence mentioned above, patients with T2DM present a high risk for heart failure, ischemic events, and CKD (24, 47).

The EMPAREG OUTCOME trial evaluated the effects of empagliflozin on CV mortality and morbidity in T2DM patients at high CV risk. Patients were randomized to receive empagliflozin or placebo once daily. This trial suggested that T2DM at high CV risk and treated with empagliflozin had a lower rate of primary outcomes than the placebo group (68).

Two years later, the CANVAS program integrated the data of 10,142 T2DM patients at high CV risk. Patients were randomized to receive canagliflozin or placebo. After a mean period of 188.2 weeks, primary outcomes were analyzed. Results showed that patients treated with canagliflozin presented a lower risk of CV events compared to the ones receiving placebo. However, the amputation rate was higher in the treatment group, but the reason is not yet fully understood (69).

The DECLARE TIMI 58 trial conducted by Wiviott et al. evaluated the CV efficacy of dapagliflozin in patients with T2DM at risk for atherosclerotic cardiovascular diseases. Participants were randomized to receive dapagliflozin or placebo. The primary outcome was a composite of major CV events (MACE) and death or hospitalization for heart failure. Secondary outcomes were a renal composite (which included ≥40% decrease in eGFR to <60 ml/min per 1.73 m^2^ of body surface area, new end-stage renal disease, or death from renal or CV causes) and death from any cause. Results from the study highlighted a lower rate of CV death or hospitalization for heart failure and renal outcome in patients treated compared to the ones treated with placebo (70). The renal outcome described in the DECLARE TIMI 58 study was not included in the original protocol so, even though encouraging, these results need to be confirmed.

Moreover, pericardial fat is involved in the establishment of coronary artery disease, especially as a concurrent condition during DM2 and obesity (42). In 2018, Sato et al. evaluated the relationship between SGLT2i and pericardial fat in 40 T2DM patients. Results showed a decrease in the pericardial fat volume, potentially caused by the improvement of systemic metabolic parameters due to the SGLT2i treatment (42). Promising results have been obtained with luseogliflozin (43).

In 2019, Perkovic et al. published the results collected from the CREDENCE study, a double-blind, randomized trial aimed to evaluate the efficacy of canagliflozin in the CV and renal protection in T2DM patients. All patients were treated with renin–angiotensin system (RAS) blockage and presented both albuminuria and an eGFR of 30 to <90 ml/min per 1.73 m^2^ of body surface area. As a result, in T2DM patients affected by kidney disease, the CV and kidney failure risk were lower in the treatment group compared to the placebo one. The median study follow-up was 2.62 years (71).

In 2020, Bhatt et al. conducted a study to evaluate the effects of sotagliflozin on CV and renal events in patients with T2DM and moderate renal impairment (SCORED). The trial aimed to verify whether sotagliflozin was non-inferior to placebo in terms of heart failure events. SCORED was a multicenter, double-blind trial that enrolled T2DM patients with chronic kidney disease and CV risk. Patients were randomized 1:1 to receive sotagliflozin or placebo. Due to a loss of funding, the trial ended up early. However, results showed that sotagliflozin was able to reduce the risk of death for CV causes, in patients with diabetes and chronic kidney disease, with or without albuminuria (47).

Interestingly, SGLT2i might change CV biomarkers in patients with CKD and T2DM. Lawler et al. performed a *post hoc* analysis among 231 participants from the eValuation of ERTugliflozin efficacy and Safety (VERTIS) renal trial affected by T2DM and stage 3 CKD. Patients were randomized 1:1 to receive ertugliflozin 5 or 15 mg daily or placebo. Clinical biomarkers have been measured at three time points: baseline, 26 weeks, and 52 weeks. The analysis included cardiac troponin, renin, N-terminal pro-B-type natriuretic peptide (NT-proBNP), atrial natriuretic peptide (ANP), human erythropoietin (EPO), ACE, ACE2, and aldosterone. Results showed that plasma aldosterone was significantly higher at 26 weeks in patients treated with ertugliflozin. However, the effect was no longer significant at 52 weeks.

On the contrary, NT-proBNP concentration was lower in patients treated with ertugliflozin at 26 and 52 weeks. Finally, the treatment with ertugliflozin did not cause any significant changes in cardiac troponin, EPO, ACE, ACE2, HbA1c, potassium, blood pressure, and eGFR. This study showed that the effects of ertugliflozin are maintained even in patients with moderate CKD (72). In VERTIS-CV, patients with T2D and micro- or macroalbuminuria were likely to benefit from HF risk reduction with an SGLT2i (73).

Recently, the EMPEROR-REDUCED trial aimed to evaluate the effects of SGLT2 inhibition with empagliflozin on major heart failure outcomes in patients with heart failure and a preserved ejection fraction. Results showed that a CV event occurred in 13.8% of the patients treated with empagliflozin and in 17.1% of patients included in the placebo group (hazard ratio, 0.79; 95% confidence interval [CI], 0.69 to 0.90; *p* < 0.001), regardless of the presence of diabetes. Moreover, the total number of hospitalizations for heart failure was lower in the treatment group compared to the placebo group (hazard ratio, 0.73; 95% CI, 0.61 to 0.88; *p* < 0.001) (74).

## SGLT2i Kidney Outcomes in Non-Diabetic Patients

Because of the benefits demonstrated in renal and CV protection, SGLT2i are likely to be incorporated as part of the guideline-directed medical therapy (GDMT) (75).

Several clinical studies have evaluated kidney outcomes in non-diabetic patients with and without heart failure.

Recently, two trials have tested dapagliflozin and empagliflozin in a population of patients affected by heart failure with reduced ejection fraction (HFrEF). In both trials, patients were in stable clinical conditions, with an NYHA class mainly between II and III, LVEF ≤ 40%, high levels of NT-proBNP, and recommended medical therapy. In both trials, patients with and without diabetes were enrolled and, among secondary endpoints, kidney outcomes were evaluated.

In DAPA HF, which enrolled 4,744 patients, during a median follow-up of 18 months, dapagliflozin (10 mg once daily), compared with placebo, significantly reduced the primary combined endpoint (76), i.e., cardiovascular death, hospitalization, or urgent visit for heart failure [16.3 vs. 21.2%, HR: 0.74 (95% CI: 0.65–0.85); *p* < 0.001]. Analogously, in EMPEROR-reduced (77), during a median follow-up of 16 months, empagliflozin (10 mg/daily) when compared with placebo significantly reduced the primary endpoint, i.e., CV deaths and heart failure hospitalizations [15.8 vs. 21%, HR: 0.75 (95% CI: 0.65–0.86); *p* < 0.001]. The favorable effect was mainly driven by the reduction of heart failure hospitalization (13.2 vs. 18.3%; HR: 0.69; 95% CI: 0.59 to 0.81), whereas no significant reduction was observed when CV death (10% vs. 10.8% HR 0.92; 95% CI: 0.75 to 1.12) and death for all causes were analyzed. In both DAPA-HF and EMPEROR-reduced, the favorable effects of SGLT2i were significant in diabetic as well as in non-diabetic patients (78).

Interestingly, in EMPEROR-reduced but not in DAPA-HF, a significantly slower decline of GFR was observed [mean slope of change in eGFR (ml/min/1.73 m^2^) per year: absolute difference 1.73 (95% CI: 1.10–2.37); *p* < 0.001]. This could be due to the lower baseline eGFR in the EMPEROR-reduced trial population (78).

In general, it is known that SGLT2i can decrease albuminuria and the risk of kidney disease progression in TD2M patients. However, Cherney et al. conducted the DIAMOND study, a randomized, double-blind, placebo-controlled, crossover trial, with the aim to evaluate the kidney effects of dapagliflozin in patients with proteinuria but without DM. Participants were enrolled among six hospitals in Canada, Malaysia, and Netherlands. Baseline characteristics included CKD, no DM diagnosis, a 24-h urinary protein excretion between >500 but ≤3500 mg, and an eGFR of at least 25 ml/min per 1.73 m^2^. All the participants were on stable renin–angiotensin system blockade (79).

Participants were randomized to firstly receive dapagliflozin 10 mg/once daily and then placebo, or *vice versa*, according to a 1:1 ratio. Each treatment lasted 6 weeks with a 6-week washout. The primary outcome was the change in the 24-h proteinuria, while secondary outcomes were changes in the measured GF (mGF), body weight, blood pressure, and the concentration of neurohormonal biomarkers (79).

Results showed that 6-week treatment with dapagliflozin did not affect proteinuria but induced a decline in mGFR and a bodyweight reduction in patients with CKD but without DM (79).

Notably, a large trial (DAPA-CKD), stopped early due to data showing benefits, has shown that the use of dapagliflozin in patients with CKD, with and without type 2 diabetes, was associated with less progression of CKD, renal mortality, and all-cause mortality. As mentioned above, the primary outcome was a composite of a sustained decline in the estimated GFR of at least 50%, end-stage kidney disease, or death from renal or cardiovascular causes. The primary outcome event occurred in 9.2% of patients treated with dapagliflozin and 14.5% of patients treated with placebo (hazard ratio, 0.61). Moreover, the hazard ratio for the composite of a sustained decline in the estimated GFR of at least 50%, end-stage kidney disease, or death from renal causes was 0.56, and the one for the composite of death from cardiovascular causes or hospitalization for heart failure was 0.71. Finally, 4.7% of the participants in the treatment group and 6.8% of the patients in the control group died. It is worth mentioning that all patients received ACE inhibitor/ARB and presented albuminuria. Thus, generalization to further categories of patients is needed. Nonetheless, this indication covers a broad range of patients with impaired kidney function and proteinuria, including patients with non-diabetic CKD (36).

## SGLT2i and Other Benefits: Focus on the Liver and Respiratory Failure in COVID-19

As reported above, SGLT2i showed numerous beneficial effects in different body locations, which extend beyond glycemic control. These beneficial effects include a reduction in body weight and blood pressure, and an improvement in uric acid concentration, oxidative stress, inflammation, and liver steatosis (80).

Also, dapagliflozin has been investigated to evaluate organ protection in high-risk patients affected by COVID-19 (81, 82). In fact, it has been hypothesized that dapagliflozin may reduce the risk of multi-organ failure and death and improve recovery in patients hospitalized with COVID-19 and have cardiometabolic risk factors (81).

In 2020, Akuta et al. conducted a retrospective study among 7 Japanese patients with non-alcoholic fatty liver disease (NAFLD) and T2DM receiving long-term treatment with canagliflozin (100 mg/day). The study aimed to determine the long-term effects of SGLT2i in NAFLD patients in the clinical outcomes and the liver histopathology (83).

During the study, liver biopsies were collected at the beginning of the trial, after 24 weeks, and after more than 1 year. As a result, the treatment with SGLT2i improved the scores of steatoses, lobular inflammation, ballooning, and fibrosis (83).

In 2020, the COVID-19 pandemic challenged the global health system since SARS-CoV-2 may lead to the development of severe pneumonia and may cause multiorgan failure. Cardiovascular and kidney complications are the most common, leading to poor outcomes, including death (82).

Since the important benefits showed by dapagliflozin in the cardio- and renal protection in patients with T2DM, the DARE-19 trial has been conducted. The study aimed to evaluate the organ protection provided by dapagliflozin in high-risk patients affected by COVID-19. DARE-19 was an international, multicenter, randomized, double-blind, placebo-control study, which involved hospitalized patients among the US, Brazil, Mexico, Argentina, India, Canada, and UK (81). Inclusion criteria included hospitalization with confirmed/suspected SARS-CoV-2 for ≤4 days, an O_2_ saturation of ≥94% on ≤5 L/min, a CXR finding c/w COVID-19, and ≥1 risk factor (HTN, Type 2 Diabetes, ASCVD, HF, and CKD). Primary endpoints consisted of preventing respiratory, cardiovascular, renal, or death events and the recovery in terms of hospitalization and clinical status in case of organ failure.

In this study, after the initial screening to confirm the COVID-19 diagnosis, patients were treated with dapagliflozin 10 mg or placebo once daily for 30 days, in addition to the local standard therapy. All patients had daily assessments until hospital discharge, death, or end of the 30-day treatment period, followed by a 60-day observational follow-up (82).

Results showed that organ dysfunction or death occurred in 11.2% of the patients treated with dapagliflozin and 13.8% of the patients included in the placebo group (hazard ratio 0.80). Moreover, 87.5% of the patients in the dapagliflozin group and 85.1% in the placebo group showed clinical status improvement (82).

In conclusion, the study showed that numerically fewer patients treated with dapagliflozin experienced organ failure and death and that dapagliflozin was well-tolerated, allowing the use of SGLT2i even in a setting of COVID-19 (82).

## SGLT2i Safety Profile and Adverse Events

SGLT2i are generally well tolerated (84). **Table 4** summarizes tolerability in the four main trials. SGLT2i present a low risk of hypoglycemia, a very fearsome and non-infrequent adverse event with some classes of hypoglycemic drugs, especially in people with diabetes with CKD (85). The reason could be the reduction of blood glucose levels by SGLT2i, which is closely linked to the amount of glucose filtered. The glucose load that goes into the pre-urine at the glomerular level depends on blood glucose and GFR. Thus, modest glycosuria is expected, as well as a milder hypoglycemic action in subjects with better glycemic compensation and impaired kidney function.

**Table 4:** Tolerability of SGLT2i across the main trials.

	EMPAREG OUTCOME	CANVAS	DECLARE-TIMI 58	CREDENCE	DAPA HF	DAPA CKD	VERTIS
Hypoglycemia	**-**	**-**	**-**	**-**	**-**	**-**	**-**
Urinary infections	**-**	**-**	**-**	**-**	**-**	**-**	**-**
Genital infection	**+**	**+**	**+**	**+**	**-**	**-**	**+**
Amputations	**-**	**+**	**-**	**-**	**-**	**-**	**-**
Fractures	**-**	**+**	**-**	**-**	**-**	**-**	**-**
Diabetic ketoacidosis	**-**	**+**	**+**	**+**	**-**	**-**	**+**
AKI	**-**	**-**	**-**	**-**	**-**	**-**	**-**

[Adapted from Costanza et al., 2020 (24)].

Urinary tract infections (UTIs) are a common side effect among SGLT2i, and they are frequently observed during treatments with other glycosuric drugs (86). In 2015, the US Food and Drug Administration (FDA) issued a notice reporting 19 cases of UTI and pyelonephritis (87). Similarly, the EMA indicated UTIs as a common adverse effect. In fact, clinical trials reported conflicting data: EMPA-REG OUTCOME, CANVAS, and DECLARE TIMI-58 did not show a significant difference in risk of UTIs compared to placebo, while in the CREDENCE canagliflozin study, it was associated with a 3-times increased risk (68–71). Moreover, a recent metanalysis that included 86 randomized clinical trials and over 50,000 subjects highlighted that SGLT2i, in relation to the risk of UTIs, are comparable to other hypoglycemic drugs (GLP-1 agonists and DDP-4 inhibitors). Compared to the placebo, canagliflozin and empagliflozin were not associated with an increased risk, while dapagliflozin showed a relative risk of 1.23 (95% CI, 1.03–1.46). In fact, secondary analyses revealed that dapagliflozin increased the risk of UTIs only at the dosage of 10 mg/day, thus outlining a dose-dependent side effect (88). According to the current evidence, the problem of UTIs is significantly reduced compared to the past. This reduction leads to several implications in clinical practice. Since DM itself is a risk factor for UTIs and their evolution is more frequently unfavorable than in non-diabetic patients (89), the fear of such adverse events and any complications could lead the clinician to significantly limit the prescription of SGLT2i. Many people with diabetes would not enjoy the cardiovascular and kidney benefits in this scenario. Realistically, further studies on populations with independent risk factors for UTIs, such as advanced age and CKD, will better clarify the safety profile of these molecules. On the other hand, educating patients to take care of personal hygiene and recognizing the suggestive symptomatology of UTIs can represent very useful interventions for prevention and prognosis (24).

Genital infections are mostly related to increased urinary glucose excretion (86). The EMPAREG OUTCOME, CANVAS, DECLARE TIMI-58, and CREDENCE trials agreed on increased risk from SGLT2i compared to placebo. In these trials, genital infections were between 4 and 9 times more frequent, although the number of events recorded was limited (68–71).

Fournier’s gangrene is a severe necrotizing infection of the external genitals and perineum that often requires a complex surgical approach, with a fatal prognosis in 7.5% of patients. Since their approval, several authors have reported cases of Fournier gangrene in subjects treated with SGLT2i (90). However, DECLARE TIMI-58, the only trial designed to assess the impact of Fournier’s gangrene, showed a reduced risk with dapagliflozin compared to placebo (70).

Surprisingly, in the CANVAS study, canagliflozin was associated with a double risk of foot and leg amputations (69). Volume depletion and increased blood viscosity have been postulated as possible responsible mechanisms. The known risk factors were the previous history of amputations, peripheral vascular disease, and neuropathy. According to the EMA request, the DECLARE TIMI-58 study collected the amputation data and concluded that there was no statistically significant difference in dapagliflozin compared to placebo (70). The increased risk has not been confirmed even in the CREDENCE, DAPA-HF, and OBSERVE-4D studies, an observational study that involved over 700,000 patients treated with the different SGLT2i (71, 75, 91).

During the CANVAS study, an increased risk of non-vertebral fractures, mainly of the limbs, emerged with canagliflozin already after 3 months of treatment. This result has not been confirmed in CANVAS-R or in DECLARE TIMI-58 (69, 70). Following retrospective studies have reported a very low or absent risk of fractures, comparable to other hypoglycemics such as GLP-1 agonists and DDP-4 inhibitors, in low-risk populations (92).

The scientific literature agrees on the increased risk of diabetic ketoacidosis associated with treatment with SGLT2i. However, a small number of events have been reported (24).

Moreover, some potential deleterious events on kidney function have been reported, such as the SGLT2i impact on electrolyte balance in exposed subjects (93).

In conclusion, SGLT2i showed a good safety profile. The knowledge of possible adverse events must guide the clinician in identifying those at increased risk and, where possible, intervene on modifiable risk factors, educate the patient to implement effective prevention, and ensure adequate follow-up for the early identification of side effect (24).

## SGLT2i: Current Guidelines and Recommendations

According to the growing evidence that highlights the benefits of SGLT2i, various scientific societies have gradually updated guidelines and acts of direction, progressively expanding the indications of using these molecules. According to the new Standards of Medical Care in Diabetes 2021 of the American Diabetes Association (ADA), metformin remains the first-line therapy in the diabetic subject, also nephropathic, without prejudice to conditions of intolerance or adverse reactions and for GFR > 30 ml/min/1.73 m^2^.

Nowadays, SGLT2i are also indicated in subjects where metformin has earned a good glycometabolic compensation, therefore even when HbA1c is targeted. In addition, whereas previously, the use of these molecules was recommended in the presence of heart failure, chronic kidney disease, and/or atherosclerotic vasculopathy, the existence of CV risk factors that pose a high risk is now sufficient. In people with diabetes with CKD, SGLT2i are preferred to GLP-1 agonists due to stronger evidence in terms of slowing the decline of GFR. In fact, GLP-1 agonists have reduced albuminuria and the risk of composite renal endpoints, while the effects on GFR are controversial (94, 95).

Since the CV and renal benefits from SGLT2i are only to a small extent attributable to the improvement of blood glucose control, it has been hypothesized that even non-diabetic subjects with CV pathology and/or MRC can benefit from this therapy (96).

In parallel with DAPA-HF, DAPA-CKD was a randomized, double-blind, placebo-controlled trial that also recruited diabetic and non-diabetic patients, as mentioned above. Arguably, obesity-induced CKD, which recognizes glomerular hyperfiltration as one of the main pathogenic factors, could benefit from the use of SGLT2i (97, 98).

In this direction, DAPA-HF was the first trial with SGLT2i to recruit non-diabetic subjects as well, assessing the effectiveness of dapagliflozin in the treatment of heart failure, in addition to the standard of care. Dapagliflozin emerged as a powerful therapy, with an excellent safety profile and a great efficacy even in subjects without DM (75). According to these results and those of the EMPEROR-reduced trial, the ESC Guidelines of the European Society of Cardiology for Heart Failure 2021 include SGLT2i as recommended therapy for patients with HFrEF (98).

Based on the currently available data, SGLT2i show important benefits at the socio-health and pharmacoeconomic levels. In fact, although their cost is higher compared to other hypoglycemics, the advantages are greater (24).

## Conclusions

Despite current measures against DM, the residual risk of ESKD, CV morbidity, and mortality remain high. SGLT2i have shown very promising results on renal outcomes and good safety profile. Therapeutic efficacy, tolerability, and costs support the broad use of these drugs in the diabetic population. In addition, SGLT2i can be widely used even in non-diabetic patients, and patients with risk factors and/or CV disease, nephropathy to different etiopathogenesis, and up to the more advanced stages of CKD. Of course, the evidence in CKD patients is still relatively limited and additional data are needed to draw a robust conclusion.

Further studies are needed to clarify any differences between different SGLT2i in CKD and between the different phenotypes (albuminuric and non-albuminuric). The SGLT2i represent another challenge to create an integrated management model and to apply therapeutic care diagnostic pathways, which lead to the involvement of a multidisciplinary team of professionals to ensure the optimization of the treatment and the follow-up management.

## Author Contributions

All authors contributed to the research, development, and content of the manuscript.

## Funding

Editorial support was provided by Edra S.p.A. and unconditionally funded by AstraZeneca. The funder was not involved in the study design, collection, analysis, interpretation of data, the writing of this article or the decision to submit it for publication.

## Conflict of Interest

MI received honoraria as a consultant in advisory boards from Astra Zeneca, Boehringer Ingelheim, Lilly, and Novartis. LG had provided research support for Abionyx and Sanofi; has served as a speaker for Fresenius, Estor, Werfen, Astellas, AstraZeneca, and Travere; and has served as a consultant for Sandoz, Sanofi, Baxter, Mundipharma, Estor, Pharmadoc, Retrophin, Travere, AstraZeneca, GSK, Novartis, and Chinook.

The remaining authors declare that the research was conducted in the absence of any commercial or financial relationships that could be construed as a potential conflict of interest.

## Publisher’s Note

All claims expressed in this article are solely those of the authors and do not necessarily represent those of their affiliated organizations, or those of the publisher, the editors and the reviewers. Any product that may be evaluated in this article, or claim that may be made by its manufacturer, is not guaranteed or endorsed by the publisher.

## References

[1] Alicic RZ Rooney MT Tuttle KR. Diabetic Kidney Disease: Challenges, Progress, and Possibilities. Clin J Am Soc Nephrol (2017) 12(12):2032–45. doi: 10.2215/CJN.11491116 PMC5718284 28522654

[2] Lozano R Naghavi M Foreman K Lim S Shibuya K Aboyans V. Global and Regional Mortality From 235 Causes of Death for 20 Age Groups in 1990 and 2010: A Systematic Analysis for the Global Burden of Disease Study 2010 [Published Correction Appears in Lancet. (2013) 381(9859):2095–128. 10.1016/S0140-6736(12)61728-0 PMC10790329 23245604

[3] Taal MW. Risk Factors and Chronic Kidney Disease. In: Skorecki K, editor. Brenner and Rector’s the Kidney, 10th Ed. Skorecki K, editor. Amsterdam: Elsevier (2015). p. 669–92.e7.

[4] Caramori ML Parks A Mauer M. Renal Lesions Predict Progression of Diabetic Nephropathy in Type 1 Diabetes. J Am Soc Nephrol (2013) 24(7):1175–81. doi: 10.1681/ASN.2012070739 PMC3699823 23687360

[5] Tyagi I Agrawal U Amitabh V Jain AK Saxena S. Thickness of Glomerular and Tubular Basement Membranes in Preclinical and Clinical Stages of Diabetic Nephropathy. Indian J Nephrol (2008) 18(2):64–9. 10.4103/0971-4065.42336 PMC2813125 20142905

[6] Fioretto P Mauer M. Histopathology of Diabetic Nephropathy. Semin Nephrol (2007) 27(2):195–207. doi: 10.1016/j.semnephrol.2007.01.012 17418688 PMC2746982

[7] Grabias BM Konstantopoulos K. The Physical Basis of Renal Fibrosis: Effects of Altered Hydrodynamic Forces on Kidney Homeostasis. Am J Physiol Renal Physiol (2014) 306(5):F473–85. doi: 10.1152/ajprenal.00503.2013 24352503

[8] Tuttle KR Bruton JL Perusek MC Lancaster JL Kopp DT DeFranzo RA. Effect of Strict Glycemic Control on Renal Hemodynamic Response to Amino Acids and Renal Enlargement in Insulin-Dependent Diabetes Mellitus [Published Correction Appears in N Engl J Med 1991 Dec 5;325(23):1666]. N Engl J Med (1991) 324(23):1626–32. doi: 10.1056/NEJM199106063242304 2030719

[9] Tuttle KR Bruton JL. Effect of Insulin Therapy on Renal Hemodynamic Response to Amino Acids and Renal Hypertrophy in Non-Insulin-Dependent Diabetes. Kidney Int (1992) 42(1):167–73. doi: 10.1038/ki.1992.274 1635346

[10] National Kidney Foundation. KDOQI Clinical Practice Guideline for Diabetes and CKD: 2012 Update. Am J Kidney Dis (2012) 60:850–86. doi: 10.1053/j.ajkd.2012.07.005 23067652

[11] Klessens CQ Woutman TD Veraar KA Zandbergen M Valk EJ Rotmans JI. An Autopsy Study Suggests That Diabetic Nephropathy Is Underdiagnosed. Kidney Int (2016) 90(1):149–56. doi: 10.1016/j.kint.2016.01.023 27165826

[12] DCCT/EDIC Research Group de Boer IH Sun W Cleary PA Lachin JM Molitch ME. Intensive Diabetes Therapy and Glomerular Filtration Rate in Type 1 Diabetes. N Engl J Med (2011) 365:2366–76. doi: 10.1056/NEJMoa1111732 PMC3270008 22077236

[13] Holman RR Paul SK Bethel MA Matthews DR Neil HAW. 10- Year Follow-Up of Intensive Glucose Control in Type 2 Diabetes. N Engl J Med (2008) 359:1577–89. doi: 10.1056/NEJMoa0806470 18784090

[14] Bilous R. Microvascular Disease: What Does the UKPDStell Us About Diabetic Nephropathy? Diabetes Med (2008) 25(Suppl 2):25–9. doi: 10.1111/j.1464-5491.2008.02496.x 18717975

[15] Retnakaran R Cull CA Thorne KI Adler AI Holman RR. UKPDS Study Group: Risk Factorsfor Renal Dysfunction in Type 2 Diabetes: U. K. Prospective Diabetes Study 74. Diabetes (2006) 55:1832–9. doi: 10.2337/db05-1620 16731850

[16] Scheen AJ. Pharmacodynamics, Efficacy, and Safety of Sodium-Glucose Co-Transporter Type 2 (SGLT2) Inhibitors for the Treatment of Type 2 Diabetes Mellitus. Drugs (2015) 75(1):33–59. doi: 10.1007/s40265-014-0337-y 25488697

[17] Wright EM Hirayama BA Loo DF. Active Sugar Transport in Health and Disease. J Intern Med (2007) 261:32–43. doi: 10.1111/j.1365-2796.2006.01746.x 17222166

[18] Gerich JE. Role of the Kidney in Normal Glucose Homeostasis and in the Hyperglycaemia of Diabetes Mellitus: Therapeutic Implications. Diabetes Med (2010) 27:136–42. doi: 10.1111/j.1464-5491.2009.02894.x PMC4232006 20546255

[19] Coady MJ Wallendor B Lapointe JY. Characterization of the Transport Activity of SGLT2/MAP17, the Renal Low-Affinity Na+-Glucose Cotransporter. Am J Physiol Renal Physiol (2017) 313:467–74. doi: 10.1152/ajprenal.00628.2016 28592437

[20] Alsahli M Gerich JE. Renal Glucose Metabolism in Normal Physiological Conditions and in Diabetes. Diabetes Res Clin Pract (2017) 133:1–9. doi: 10.1016/j.diabres.2017.07.033 28866383

[21] Helal I Fick-Brosnahan GM Reed-Gitomer B Schrier RW. Glomerular Hyperfiltration: Definitions, Mechanisms, and Clinical Implications. Nat Rev Nephrol (2012) 8(5):293–300. doi: 10.1038/nrneph.2012.19 22349487

[22] Zhang Y Nakano D Guan Y Hitomi H Uemura A Masaki T. A Sodium-Glucose Cotransporter 2 Inhibitor Attenuates Renal Capillary Injury and Fibrosis by a Vascular Endothelial Growth Factor-Dependent Pathway After Renal Injury in Mice. Kidney Int (2018) 94(3):524–35. doi: 10.1016/j.kint.2018.05.002 30045814

[23] Pugliese G. Updating the Natural History of Diabetic Nephropathy. Acta Diabetol (2014) 51(6):905–15. doi: 10.1007/s00592-014-0650-7 25297841

[24] Costanza G Pesce F Forcella M Leonardi G Seminara G Di Natale E. SGLT2 Inibitori, Non Solo Ipoglicemizzanti: Impatto Nella Pratica Clinica Nefrologica. G Ital Nefrol (2020) 4(2):1–19.

[25] Bonnet F Scheen AJ. SGLT-2 Inhibitors: An Opportunity to Renew Our Therapeutic Strategy for Type 2 Diabetes? Diabetes Metab (2014) 40(Suppl):S1–3. doi: 10.1016/S1262-3636(14)72688-6 25554065

[26] Neumiller JJ White JR Jr Campbell RK. Sodium-Glucose Cotransport Inhibitors: Progress and Therapeutic Potential in Type 2 Diabetes Mellitus. Drugs (2010) 70(4):377–85. doi: 10.2165/11318680-000000000-00000 20205482

[27] Tahrani AA Barnett AH Bailey CJ. SGLT Inhibitors in Management of Diabetes. Lancet Diabetes Endocrinol (2013) 1(2):140–51. doi: 10.1016/S2213-8587(13)70050-0 24622320

[28] Hasan FM Alsahli M Gerich JE. SGLT2 Inhibitors in the Treatment of Type 2 Diabetes. Diabetes Res Clin Pract (2014) 104(3):297–322. doi: 10.1016/j.diabres.2014.02.014 24735709

[29] Bailey CJ. Renal Glucose Reabsorption Inhibitors to Treat Diabetes. Trends Pharmacol Sci (2011) 32(2):63–71. doi: 10.1016/j.tips.2010.11.011 21211857

[30] Abdul-Ghani MA Norton L Defronzo RA. Role of Sodiumglucose Cotransporter 2 (SGLT 2) Inhibitors in the Treatment of Type 2 Diabetes. Endocr Rev (2011) 32(4):515–31. doi: 10.1210/er.2010-0029 21606218

[31] Scheen AJ Paquot N. Metabolic Effects SGLT2 Inhibitors Beyond Increased Glucosuria: A Review of Clinical Evidence. Diabetes Metab (2014) 40(Suppl):S4–11. doi: 10.1016/S1262-3636(14)72689-8 25554070

[32] Takebayashi K Inukai T. Effect of Sodium Glucose Cotransporter 2 Inhibitors With Low SGLT2/SGLT1 Selectivity on Circulating Glucagon-Like Peptide 1 Levels in Type 2 Diabetes Mellitus. J Clin Med Res (2017) 9(9):745–53. doi: 10.14740/jocmr3112w PMC5544478 28811850

[33] Zelniker TA Wiviott SD Raz I Im K Goodrich EL Bonaca MP. SGLT2 Inhibitors for Primary and Secondary Prevention of Cardiovascular and Renal Outcomes in Type 2 Diabetes: A Systematic Review and Meta-Analysis of Cardiovascular Outcome Trials [Published Correction Appears in Lancet 2019 Jan 5;393(10166):30]. Lancet (2019) 393(10166):31–9. doi: 10.1016/S0140-6736(18)32590-X 30424892

[34] Kluger AY Tecson KM Lee AY Lerma EV Rangaswami J Lepor NE. Class Effects of SGLT2 Inhibitors on Cardiorenal Outcomes. Cardiovasc Diabetol (2019) 18(1):99. doi: 10.1186/s12933-019-0903-4 31382965 PMC6683461

[35] Toyama T Neuen BL Jun M Ohkuma T Neal B Jardine MJ. Effect of SGLT2 Inhibitors on Cardiovascular, Renal and Safety Outcomes in Patients With Type 2 Diabetes Mellitus and Chronic Kidney Disease: A Systematic Review and Meta-Analysis. Diabetes Obes Metab (2019) 21(5):1237–50. doi: 10.1111/dom.13648 30697905

[36] Heerspink HJL Stefánsson BV Correa-Rotter R Chertow GM Greene T Hou F-F. Dapagliflozin in Patients With Chronic Kidney Disease. N Engl J Med (2020) 383:1436–46. doi: 10.1056/NEJMoa2024816 32970396

[37] O’Brien TP Jenkins EC Estes SK Castaneda AV Ueta K Farmer TD. Correcting Postprandial Hyperglycemia in Zucker Diabetic Fatty Rats With an SGLT2 Inhibitor Restores Glucose Effectiveness in the Liver and Reduces Insulin Resistance in Skeletal Muscle. Diabetes (2017) 66(5):1172–84. doi: 10.2337/db16-1410 PMC5399614 28246292

[38] Merovci AC Solis-Herrera C Daniele G Eldor R Fiorentino TV Tripathy D. Dapagliflozin Improves Muscle Insulin Sensitivity But Enhances Endogenous Glucose Production. J Clin Invest (2014) 124(2):509–14. doi: 10.1172/JCI70704 PMC3904617 24463448

[39] Vasilakou D Karagiannis T Athanasiadou E Mainou M Liakos A Bekiari E. Sodium-Glucose Cotransporter 2 Inhibitors for Type 2 Diabetes: A Systematic Review and Meta-Analysis. Ann Intern Med (2013) 159(4):262–74. doi: 10.7326/0003-4819-159-4-201308200-00007 24026259

[40] Heerspink HJL Desai M Jardine M Balis D Meininger G Perkovic V. Canagliflozin Slows Progression of Renal Function Decline Independently of Glycemic Effects. J Am Soc Nephrol (2017) 28(1):368–75. doi: 10.1681/ASN.2016030278 PMC5198289 27539604

[41] Yagi S Hirata Y Ise T Kasunose K Yamada H Fukuda D. Canagliflozin Reduces Epicardial Fat in Patients with Type 2 Diabetes Mellitus. Diabetol Metab Syndr (2017) 9:78. doi: 10.1186/s13098-017-0275-4 29034006 PMC5628447

[42] Sato T Aizawa Y Yuasa S Kishi S Fuse K Fujita S. The Effect of Dapagliflozin Treatment on Epicardial Adipose Tissue Volume. Cardiovasc Diabetol (2018) 17(1):6. doi: 10.1186/s12933-017-0658-8 29301516 PMC5753537

[43] Bouchi R Terashima M Sasahara Y Asakawa M Fukuda T Takeuchi T. Luseogliflozin Reduces Epicardial Fat Accumulation in Patients With Type 2 Diabetes: A Pilot Study. Cardiovasc Diabetol (2017) 16(1):32. doi: 10.1186/s12933-017-0516-8 28253918 PMC5335851

[44] Blonde L Stenlöf K Fung A Xie J Canovatchel W Meininger G. Effects of Canagliflozin on Body Weight and Body Composition in Patients With Type 2 Diabetes Over 104 Weeks. Postgrad Med (2016) 128(4):371–80. doi: 10.1080/00325481.2016.1169894 27002421

[45] McGurnaghan SJ Brierley L Caparrotta TM McKeigue PM Blackbourn LAK Wild SH. The Effect of Dapagliflozin on Glycaemic Control and Other Cardiovascular Disease Risk Factors in Type 2 Diabetes Mellitus: A Real-World Observational Study. Diabetologia (2019) 62(4):621–32. doi: 10.1007/s00125-018-4806-9 30631892

[46] Kario K Okada K Kato M Nishizama M Yoshida T Asano T. 24-Hour Blood Pressure-Lowering Effect of an SGLT-2 Inhibitor in Patients With Diabetes and Uncontrolled Nocturnal Hypertension: Results From the Randomized, Placebo-Controlled SACRA Study. Circulation (2018) 139(18):2089–97. doi: 10.1161/CIRCULATIONAHA.118.037076 PMC6493695 30586745

[47] Bhatt DL Szarek M Pitt B Cannon CP Leiter LA McGuire DK. Sotagliflozin in Patients With Diabetes and Chronic Kidney Disease. N Engl J Med (2021) 384(2):129–39. doi: 10.1056/NEJMoa2030186 33200891

[48] Lee PC Ganguly S Goh SY. Weight Loss Associated With Sodium-Glucose Cotransporter-2 Inhibition: A Review of Evidence and Underlying Mechanisms. Obes Rev (2018) 19(12):1630–41. doi: 10.1111/obr.12755 30253050

[49] Schork A Saynisch J Vosseler A Jaghutriz BA Heyne N Peter A. Effect of SGLT2 Inhibitors on Body Composition, Fluid Status and Renin-Angiotensin-Aldosterone System in Type 2 Diabetes: A Prospective Study Using Bioimpedance Spectroscopy. Cardiovasc Diabetol (2019) 18(1):46. doi: 10.1186/s12933-019-0852-y 30953516 PMC6451223

[50] Hou Y-C Zheng C-M Yen T-H Kuo-Cheng Lu K-C. Molecular Mechanisms of SGLT2 Inhibitor on Cardiorenal Protection. Int J Mol Sci (2020) 21(21):7833. doi: 10.3390/ijms21217833 33105763 PMC7660105

[51] Verma S McMurray JJV. SGLT2 Inhibitors and Mechanisms of Cardiovascular Benefit: A State-of-the-Art Review. Diabetologia (2018) 61(10):2108–17. doi: 10.1007/s00125-018-4670-7 30132036

[52] Hallow KH Helmlinger G Greasley PJ McMurray JJV Boulton DW. Why do SGLT2 Inhibitors Reduce Heart Failure Hospitalization? A Differential Volume Regulation Hypothesis. Diabetes Obes Metab (2018) 20(3):479–87. doi: 10.1111/dom.13126 29024278

[53] Griffin M Rao VS Ivey-Miranda J Fleming J Mahoney D Maulion C. Empagliflozin in Heart Failure. Diuretic and Cardiorenal Effects. Circulation (2020) 142:1028–39. doi: 10.1161/CIRCULATIONAHA.120.045691 PMC7521417 32410463

[54] DeFronzo RA Norton L Abdul-Ghani M. Renal, Metabolic, and Cardiovascular Considerations of SGLT2 Inhibition. Nat Rev Nephrol (2017) 13(1):11–26. doi: 10.1038/nrneph.2016.170 27941935

[55] Eickhoff MK Dekkers CCJ Kramers BJ Laverman GD Frimodt-Møller M Jørgensen NR. Effects of Dapagliflozin on Volume Status When Added to Renin-Angiotensin System Inhibitors. J Clin Med (2019) 8(6):779. doi: 10.3390/jcm8060779 31159350 PMC6616433

[56] Heerspink HJ de Zeeuw D Wie L Leslie B List J. Dapagliflozin a Glucose-Regulating Drug With Diuretic Properties in Subjects With Type 2 Diabetes. Diabetes Obes Metab (2013) 15(9):853–62. doi: 10.1111/dom.12127 PMC3906841 23668478

[57] Karg MV Bosch A Kannenkeril D Striepe K Ott C Schneider MP. SGLT-2-Inhibition With Dapagliflozin Reduces Tissue Sodium Content: A Randomised Controlled Trial. Cardiovasc Diabetol (2018) 17(1):5. doi: 10.1186/s12933-017-0654-z 29301520 PMC5753452

[58] Solini A Seghieri M Giannini L Biancalana E Parolini F Rossi C. The Effects of Dapagliflozin on Systemic and Renal Vascular Function Display an Epigenetic Signature. J Clin Endocrinol Metab (2019) 104(10):4253–63. doi: 10.1210/jc.2019-00706 31162549

[59] Wang XX Levi J Luo Y Myakala K Herman-Edelstein M Qiu L. SGLT2 Protein Expression Is Increased in Human Diabetic Nephropathy: SGLT2 Protein Inhibition Decreases Renal Lipid Accumulation, Inflammation, Inflammation, and the Development of Nephropathy in Diabetic MICE. J Biol Chem (2017) 292(13):5335–48. doi: 10.1074/jbc.M117.779520 PMC5392679 28196866

[60] Inzucchi SE Zinman B Fitchett D Wanner C Ferrannini E Schumacher M. How Does Empagliflozin Reduce Cardiovascular Mortality? Insights From a Mediation Analysis of the EMPA-REG OUTCOME Trial. Diabetes Care (2018) 41(2):356–63. doi: 10.2337/dc17-1096 29203583

[61] Ferrannini E Baldi S Frascerra S Astiarraga B Heise T Bizzotto R. Shift to Fatty Substrate Utilization in Response to Sodium-Glucose Cotransporter 2 Inhibition in Subjects Without Diabetes and Patients With Type 2 Diabetes. Diabetes (2016) 65(5):1190–5. doi: 10.2337/db15-1356 26861783

[62] Sano M Takei M Shiraishi Y Suzuki Y. Increased Hematocrit During Sodium-Glucose Cotransporter 2 Inhibitor Therapy Indicates Recovery of Tubulointerstitial Function in Diabetic Kidneys. J Clin Med Res (2016) 8(12):844–7. doi: 10.14740/jocmr2760w PMC5087622 27829948

[63] Ghanim H Abuaysheh S Hejna J Green K Batra M Makdissi A. Dapagliflozin Suppresses Hepcidin And Increases Erythropoiesis. J Clin Endocrinol Metab (2020) 105(4):dgaa057. doi: 10.1210/clinem/dgaa057 32044999

[64] Cassis P Locatelli M Cerullo D Corna D Buelli S Zanchi C. SGLT2 Inhibitor Dapagliflozin Limits Podocyte Damage in Proteinuric Nondiabetic Nephropathy. JCI Insight (2018) 3:98720. doi: 10.1172/jci.insight.98720 30089717 PMC6129124

[65] Wheeler DC Toto RD Stefánsson BV Jongs N Chertow GM Greene T. A Pre-Specified Analysis of the DAPA-CKD Trial Demonstrates the Effects of Dapagliflozin on Major Adverse Kidney Events in Patients With IgA Nephropathy. Kidney Int (2021) 100:215–24. doi: 10.1016/j.kint.2021.03.033 33878338

[66] De Nicola L Gabbai FB Liberti ME Sagliocca A Conte G Minutolo R. Sodium/glucose Cotransporter 2 Inhibitors and Prevention of Diabetic Nephropathy: Targeting the Renal Tubule in Diabetes. Am J Kidney Dis (2014) 64(1):16–24. doi: 10.1053/j.ajkd.2014.02.010 24673844

[67] FDA Approves Treatment for Chronic Kidney Disease. Available at: www.fda.gov/news-events/press-announcements/fda-approves-treatment-chronic-kidney-disease.

[68] Zinman B Wanner C Lachin JM Fitchett D Bluhmki E Hantel S. Empagliflozin, Cardiovascular Outcomes, and Mortality in Type 2 Diabetes. N Engl J Med (2015) 373(22):2117–28. doi: 10.1056/NEJMoa1504720 26378978

[69] Neal B Perkovic V Mahaffey Fitchett D Bluhmki E Hantel S. Canagliflozin and Cardiovascular and Renal Events in Type 2 Diabetes. N Engl J Med (2017) 377(7):644–57. doi: 10.1056/NEJMc1712572 28605608

[70] Wiviott SD Raz I Bonaca MP Mosenzon O Kato ET Cahn A. Dapagliflozin and Cardiovascular Outcomes in Type 2 Diabetes. N Engl J Med (2019) 380(4):347–57. doi: 10.1056/NEJMoa1812389 30415602

[71] Perkovic V Jardine MJ Neal B Bompoint S Heerspink HJL Charytan DM. Canagliflozin and Renal Outcomes in Type 2 Diabetes and Nephropathy. N Engl J Med (2019) 380(24):2295–306. doi: 10.1056/NEJMoa1811744 30990260

[72] Lawler PR Liu H Frankfurter C Lovblom LE Lytvyn Y Burger D. Changes in Cardiovascular Biomarkers Associated With the Sodium-Glucose Cotransporter 2 (SGLT2) Inhibitor Ertugliflozin in Patients With Chronic Kidney Disease and Type 2 Diabetes. Diabetes Care (2021) 44(3):e45–7. doi: 10.2337/dc20-2265 33436398

[73] Cosentino F Cannon CP Cherney DZI Masiukiewicz U Pratley R Dagogo-Jack S. Efficacy of Ertugliflozin on Heart Failure-Related Events in Patients With Type 2 Diabetes Mellitus and Established Atherosclerotic Cardiovascular Disease: Results of the VERTIS CV Trial. Circulation (2020) 14(23):22205–2215. doi: 10.1161/CIRCULATIONAHA.120.050255 PMC7717477 33026243

[74] Anker SD Butler J Filippatos G Ferreira JP Bocchi E Böhm M. Empagliflozin in Heart Failure With a Preserved Ejection Fraction. N Engl J Med (2021) 385(16):1451–61. doi: 10.1056/NEJMoa2107038 34449189

[75] McMurray JJV Solomon SD Inzucchi SE Kober L Kosiborod MN Martinez FA. Dapagliflozin in Patients With Heart Failure and Reduced Ejection Fraction. N Engl J Med (2019) 381:1995–2008. doi: 10.1056/NEJMoa1911303 31535829

[76] McMurray JJV Packer M. How Should We Sequence the Treatments for Heart Failure and a Reduced Ejection Fraction? Circulation (2021) 143:875–7. doi: 10.1161/CIRCULATIONAHA.120.052926 33378214

[77] Packer M Anker SD Butler J Filippatos G Pocock SJ Carson P. Cardiovascular and Renal Outcomes With Empagliflozin in Heart Failure. N Engl J Med (2020) 383:1413–24. doi: 10.1056/NEJMoa2022190 32865377

[78] Zannad F Ferreira JP Pocock SJ Anker SD Butler J Filippatos G. SGLT2 Inhibitors in Patients With Heart Failure With Reduced Ejection Fraction: A Meta-Analysis of the EMPEROR-Reduced and DAPA-HF Trials. Lancet (2020) 396(10254):P819–829. doi: 10.1016/S0140-6736(20)31824-9 32877652

[79] Cherney DZI Dekkers CCJ Barbour SJ Cattran D Abdul Gafor AH Greasley PJ. Effects of the SGLT2 Inhibitor Dapagliflozin on Proteinuria in non-Diabetic Patients With Chronic Kidney Disease (DIAMOND): A Randomised, Double-Blind, Crossover Trial [Published Correction Appears in Lancet Diabetes Endocrinol 2020 Jun 25]. Lancet Diabetes Endocrinol (2020) 8(7):582–93. doi: 10.1016/S2213-8587(20)30162-5 32559474

[80] Bonora E Cataudella S Marchesini G Miccoli R Vaccaro O Fadini GP. A View on the Quality of Diabetes Care in Italy and the Role of Diabetes Clinics From the 2018 ARNO Diabetes Observatory. Nutr Metab Cardiovasc Dis (2020) 30(11):1945–53. doi: 10.1016/j.numecd.2020.08.018 32998821

[81] Kosiborod M Berwanger O Koch GG Martinez F Mukhtar O Verma S. Effects of Dapagliflozin on Prevention of Major Clinical Events and Recovery in Patients With Respiratory Failure Because of COVID-19: Design and Rationale for the DARE-19 Study. Diabetes Obes Metab (2021) 23(4):886–96. doi: 10.1111/dom.14296 PMC8049025 33319454

[82] Kosiborod MN Esterline R Furtado RHM Oscarsson J Gasparyan SB Koch GG. Dapagliflozin in Patients With Cardiometabolic Risk Factors Hospitalised With COVID-19 (DARE-19): A Randomised, Double-Blind, Placebo-Controlled, Phase 3 Trial. Lancet Diabetes Endocrinol (2021) 9(9):586–94. doi: 10.1016/S2213-8587(21)00180-7 PMC8294807 34302745

[83] Akuta N Kawamura Y Fujiyama S Sezaki H Hosaka T Kobayashi M. SGLT2 Inhibitor Treatment Outcome in Nonalcoholic Fatty Liver Disease Complicated With Diabetes Mellitus: The Long-Term Effects on Clinical Features and Liver Histopathology. Intern Med (2020) 59(16):1931–7. doi: 10.2169/internalmedicine.4398-19 PMC7492114 32448832

[84] Zelniker TA Wiviott SD Raz I Im K Goodrich EL Bonaca MP. SGLT2 Inhibitors for Primary and Secondary Prevention of Cardiovascular and Renal Outcomes in Type 2 Diabetes: A Systematic Review and Meta-Analysis of Cardiovascular Outcome Trials. Lancet (2019) 393:31–9. doi: 10.1016/S0140-6736(18)32590-X 30424892

[85] Moen MF Zhan M Hsu VD Walker LD Einhorn LM Seliger SL. Frequency of Hypoglycemia and Its Significance in Chronic Kidney Disease. Clin J Am Soc Nephrol (2009) 4:1121–27. doi: 10.2215/CJN.00800209 PMC2689888 19423569

[86] Geerlings S Fonseca V Castro-Diaz D List J Parikh S. Genital and Urinary Tract Infections in Diabetes: Impact of Pharmacologically-Induced Glucosuria. Diabetes Res Clin Pract (2014) 103(3):373–81. doi: 10.1016/j.diabres.2013.12.052 24529566

[87] S. Food and Drug Administration. FDA Drug Safety Communication: FDA Revises Labels of SGLT2 Inhibitors for Diabetes to Include Warnings About Too Much Acid in the Blood and Serious Urinary Tract Infections. (2015).

[88] Puckrin R Saltiel MP Reynier P Azoulay L Yu OHY Filion KB. SGLT-2 Inhibitors and the Risk of Infections: A Systematic Review and Meta-Analysis of Randomized Controlled Trials. Acta Diabetol (2018) 55(5):503–14. doi: 10.1007/s00592-018-1116-0 29484489

[89] Nitzan O Elias M Chazan B Saliba W. Urinary Tract Infections in Patients With Type 2 Diabetes Mellitus: Review of Prevalence, Diagnosis, and Management. Diabetes Metab Syndr Obes (2015) 8:129–36. doi: 10.2147/DMSO.S51792 PMC4346284 25759592

[90] Bersoff-Matcha SJ Chamberlain C Cao C Kortepeter C Chong WH. Fournier Gangrene Associated With Sodium-Glucose Cotransporter-2 Inhibitors: A Review of Spontaneous Postmarketing Cases. Ann Intern Med (2019) 170(11):764–9. doi: 10.7326/M19-0085 31060053

[91] Ryan P Buse JB Schuemie M Defalco F Yuan Z Stang P. Canagliflozin (CANA) vs. Other Antihyperglycemic Agents on the Risk of Below-Knee Amputation (BKA) for Patients With T2DM—A Real-World Analysis of >700,000 U.S. Patients. Diabetes (2018) 67:4. doi: 10.2337/db18-4-LB. 67(Supplement 1).

[92] Fralick M Kim SC Schneeweiss S Kim D Redelmeier DA Patorno E. Fracture Risk After Initiation of Use of Canagliflozin: A Cohort Study. Ann Intern Med (2019) 170(3):155–63. doi: 10.7326/M18-0567 PMC6602870 30597484

[93] Cianciolo G De Pascalis A Capelli I Gasperoni L Di Lullo L Bellasi A. Mineral and Electrolyte Disorders With SGLT2i Therapy. JBMR Plus (2019) 3(11):e10242. doi: 10.1002/jbm4.10242 31768494 PMC6874177

[94] American Diabetes Association. Standards of Medical Care in Diabetes-2020 Abridged for Primary Care Providers. Clin Diabetes (2020) 38(1):10–38. doi: 10.2337/cd20-as01 31975748 PMC6969656

[95] Giugliano D Maiorino MI Bellastella G Longo M Chiodini P Esposito K. GLP-1 Receptor Agonists for Prevention of Cardiorenal Outcomes in Type 2 Diabetes: An Updated Meta-Analysis Including the REWIND and PIONEER 6 Trials. Diabetes Obes Metab (2019) 21(11):2576–80. doi: 10.1111/dom.13847 31373167

[96] Dekkers CCJ Gansevoort RT. Sodium-Glucose Cotransporter 2 Inhibitors: Extending the Indication to non-Diabetic Kidney Disease? Nephrol Dial Transplant (2020) 35(Suppl 1):i33–42. doi: 10.1093/ndt/gfz264 PMC6993196 32003836

[97] Chagnac A Zingerman B Rozen-Zvi B Herman-Edelstein M. Consequences of Glomerular Hyperfiltration: The Role of Physical Forces in the Pathogenesis of Chronic Kidney Disease in Diabetes and Obesity. Nephron (2019) 143(1):38–42. doi: 10.1159/000499486 30947190

[98] Visseren FLJ Mach F Smulders YM Carballo D Koskinas KC Bäck M. 2021 ESC Guidelines on Cardiovascular Disease Prevention in Clinical Practice: Developed by the Task Force for Cardiovascular Disease Prevention in Clinical Practice With Representatives of the European Society of Cardiology and 12 Medical Societies With the Special Contribution of the European Association of Preventive Cardiology (EAPC). Eur Heart J (2021) 42(34):3227–337. doi: 10.1093/eurheartj/ehab484

